# Barriers and facilitators to breastfeeding support practices in a neonatal intensive care unit in Colombia

**DOI:** 10.17533/udea.iee.v39n1e11

**Published:** 2021-03-05

**Authors:** Haley Abugov, Sandra Catalina Ochoa Marín, Sonia Semenic, Isabel Cristina Arroyave

**Affiliations:** 1 R.N., M.Sc.(A), Montreal Children’s Hospital, McGill University Health Center (Canada). Email: haley.abugov@muhc.mcgill.ca McGill University Montreal Children’s Hospital, McGill University Health Center Canada haley.abugov@muhc.mcgill.ca; 2 R.N., Ph.D. University of Antioquia, Faculty of Nursing, Medellín (Colombia). Email: catalina.ochoa@udea.edu.co Universidad de Antioquia University of Antioquia Medellín Colombia catalina.ochoa@udea.edu.co; 3 R.N., Ph.D. McGill University, Ingram School of Nursing (Canada). Email: sonia.semenic@mcgill.ca McGill University McGill University, Ingram School of Nursing Canada sonia.semenic@mcgill.ca; 4 R.N., Ph.D. Hospital Universitario San Vicente Fundación, Medellín (Colombia). Email: isaarroyave@hotmail.com Hospital Universitario San Vicente Fundación Medellín Colombia isaarroyave@hotmail.com

**Keywords:** infant, newborn, breast feeding, intensive care units, neonatal, milk, human, quality improvement, recién nacido, cuidado intensivo neonatal, lactancia materna, leche humana, mejoramiento de la calidad, recém-nascido, terapia intensiva neonata, aleitamento materno, leite humano, melhoria de qualidade

## Abstract

**Objective.:**

To assess breastfeeding support practices and related barriers and facilitators in a large Intensive Care Unit, Neonatal (NICU) in Medellín, Colombia, as part of a broader quality improvement initiative to enhance breastfeeding support.

**Methods.:**

A mixed-methods descriptive design was used to collect data on care practices and outcomes related to NICU breastfeeding support. Data sources included the Neo-BFHI’s self-assessment questionnaire of breastfeeding policies and practices, clinical observations, and a retrospective review of 51 patient charts.

**Results.:**

Of the 51 charts reviewed, 98% of the infants received breastmilk during their hospitalization but the majority (84%) also received formula and only 8% of infants were exclusively breastfed at the time of NICU discharge. All NICU staff received education on mother and baby-friendly care, and the unit complied with the International Code of Marketing of Breast-milk substitutes. However, resources to support lactation (e.g., access to breastfeeding specialists, breast pumps, written teaching materials for parents) were limited, and infants were only allowed to consume milk expressed within the hospital. Mother-infant separation, as well as staff beliefs and care routines, also limited important breastfeeding support practices such as skin-to-skin care and early initiation of direct breastfeeding.

**Conclusion.:**

The self-assessment questionnaire and observations revealed a high value for breastfeeding and a family-centered approach to care in the NICU. Key challenges to sustaining breastfeeding in the NICU included a lack of facilities for supporting parental presence, barriers to expression and provision of mother’s milk, and a high rate of bottle-feeding with formula.

## Resumen

**Objetivo.:**

valuar las prácticas de apoyo a la lactancia materna y las barreras y los facilitadores relacionados en una gran unidad de cuidados intensivos neonatal (UCIN) de Medellín, Colombia.

**Métodos.:**

Se utilizó un diseño descriptivo de métodos mixtos para recopilar información sobre las prácticas de atención y los resultados relacionados con el apoyo a la lactancia materna en la UCIN. Las fuentes de datos incluyeron la encuesta de autoevaluación de las políticas y las prácticas de lactancia materna de Neo-BFHI, observaciones clínicas y una revisión retrospectiva de las historias clínicas de pacientes.

**Resultados.:**

De las 51 historias clínicas revisadas, el 98% de los bebés recibieron leche materna durante su hospitalización, pero la mayoría (84%) también recibió fórmula y solo el 8% de los bebés fueron amamantados exclusivamente al momento del alta de la UCIN. Todo el personal recibió educación sobre cuidado amigable a las madres y sus bebés, y la unidad cumplió con el Código Internacional de Comercialización de Sustitutos de la Leche Materna. Sin embargo, los recursos para apoyar la lactancia (por ejemplo, el acceso a especialistas, extractores de leche, material didáctico escrito para los padres) fueron limitados y los bebés solo podían consumir leche extraída dentro del hospital. La separación madre-hijo, así como las creencias del personal y las rutinas de atención, también limitaron prácticas importantes de apoyo como el cuidado piel con piel y el inicio temprano de la lactancia materna directa.

**Conclusión.:**

El cuestionario de autoevaluación y las observaciones revelaron un alto valor de la lactancia materna y un enfoque de atención centrado en la familia en la UCIN. Los desafíos clave incluyeron la falta de instalaciones para apoyar la presencia de los padres, las barreras para la extracción y el suministro de leche y una alta tasa de alimentación con biberón con fórmula.

## Resumo

**Objetivo.:**

Avaliar as práticas de apoio à amamentação e as barreiras e facilitadores relacionados em uma grande Unidade de Terapia Intensiva Neonatal -UTIN- em Medellín, Colômbia.

**Métodos.:**

Um desenho descritivo de métodos mistos foi usado para coletar informações sobre as práticas de cuidado e resultados relacionados ao apoio à amamentação na UTIN. As fontes de dados incluíram a Pesquisa de Autoavaliação de Políticas e Práticas de Amamentação da Neo-IHAC, observações clínicas e uma revisão retrospectiva dos registros dos pacientes.

**Resultados.:**

Dos 51 prontuários analisados, 98% dos bebês receberam leite materno durante a internação, mas a maioria (84%) também recebeu fórmula e apenas 8% dos bebês foram amamentados exclusivamente na alta da UTIN. Todos os funcionários da UTIN receberam educação sobre cuidados amigáveis para mães e bebês, e a unidade estava em conformidade com o Código Internacional de Comercialização de Substitutos do Leite Materno. No entanto, os recursos para apoiar a amamentação (por exemplo, acesso a especialistas em amamentação, extratores de leite, materiais de treinamento escritos para os pais) eram limitados e os bebês só podiam consumir leite extraído dentro do hospital. As separações mães-bebês, bem como as crenças da equipe e rotinas de cuidado, também limitaram práticas importantes de apoio à amamentação, como cuidados pele a pele e início precoce da amamentação direta.

**Conclusão.:**

O questionário de autoavaliação e as observações revelaram alto valor para a amamentação e uma abordagem centrada na família para o cuidado na UTIN. Os principais desafios para manter a amamentação na UTIN incluíram a falta de instalações para apoiar a presença dos pais, barreiras para extrair e fornecer leite materno e uma alta taxa de alimentação com mamadeira com fórmula.

## Introduction

Breastfeeding is a global public health priority. The World Health Organization’s (WHO) *Global Strategy for Infant and Young Child Feeding* recommends exclusive breastfeeding for the first six months of life and continued breastfeeding for up to two years and beyond to support optimal infant growth and development.(1) In 2010, Colombia’s Ministry of Social Protection, and Ministry of National Education, launched The Ten-Year Breastfeeding Plan (2010-2020) to address the fundamental importance of breastfeeding for the well-being and quality of life of children.(2) The plan outlined five key strategies (e.g., support, promotion, social mobilization) as well as three main objectives, including the development of institutional capacities that support, promote and protect breastfeeding.(2) However, breastfeeding rates in Colombia remain well below the WHO recommendations, with a median duration of exclusive breastfeeding of only 1.8 months and less than 60% of infants still breastfeeding at 1 year.(3)

The benefits of human milk for infants are well-documented and are especially important for ill or premature infants hospitalized in neonatal intensive care units (NICU).(4) Feeding breastmilk to premature or ill infants provides significant nutritional, gastrointestinal, immunological, developmental and psychological benefits. These include lower rates of nosocomial infections, sepsis, necrotizing enterocolitis, and severe retinopathy of prematurity; promotion of maternal-infant attachment; earlier NICU discharge and decreased rates of re-hospitalization for illness in the first year following NICU discharge.(4-6) The Baby-Friendly Hospital Initiative (BFHI) is a global strategy established by the WHO and UNICEF that provides healthcare facilities with a framework to improve practices that promote, protect and support breastfeeding.(7) A 2017 survey of 117 countries found that 86% had implemented the BFHI.(8) Colombia adapted the BFHI to create their own “breastfeeding-friendly” certification program - the IAMI (Women and Child Friendly Institutions).(9) In 2013, the BFHI was expanded into a new international program, the “Neo-BFHI”, to address the special breastfeeding support needs of preterm and ill infants hospitalized in NICUs. In addition to adapting the original BFHI’s “Ten Steps to Successful Breastfeeding” for healthy term birth infants, the Neo-BFHI added Three Guiding Principles.(10) These guiding principles include focusing on the mother’s individualized needs; providing family-centered care and ensuring continuity of care from prenatal to post discharge. The Neo-BFHI’s adapted Ten Steps address NICU practices and policies, staff education and organization of the physical environment and provide specific recommendations for supporting the initiation, continuation and exclusivity of breastfeeding (10). The Neo-BFHI program also includes a self-assessment tool for hospitals to measure their compliance with the Neo-BFHI’s Ten Steps and Three Guiding Principles. This tool was recently integrated into an online questionnaire and translated into multiple languages, as part of a large international survey of breastfeeding policies and practices in neonatal wards.(11)

The busy, stressful NICU environment presents many challenges to successful implementation of Baby-friendly practices.(4) Rates of breastfeeding initiation and duration among infants in the NICU are lower than among healthy infants born full-term.(12) Studies from high-income countries have described numerous barriers to the establishment of breastfeeding in the NICU, including medical fragility and other physical challenges that may interfere with infant feeding, maternal distress related to the infant’s hospitalization, physical separation of mothers and infants, lack of privacy, and inconsistent breastfeeding support.(13-16) Other common challenges to breastfeeding faced by mothers include rigid feeding schedules and strict monitoring of infant intake in the NICU, delayed initiation of milk expression or difficulties maintaining adequate milk volumes until the infant is capable of feeding at the breast, and difficulty transitioning the baby from gavage to breastfeeding.(13-16) Whereas similar obstacles to breastfeeding in the NICU have been reported in studies from Brazil, (17^,^^18^) no studies were found on breastfeeding practices and outcomes among ill or preterm infants hospitalized in neonatal units in Colombia.

Although not specifically focused on breastfeeding, a previous study exploring staff nurse perceptions of parental readiness for NICU discharge at the Hospital Universitario San Vicente Fundación (HUSVF) in Medellin (Colombia) identified breastfeeding challenges as an important concern related to infant discharge home.(19) Key obstacles to successful breastfeeding reported by the NICU staff included difficulties maintaining milk production, mother-infant separation, and hospital policies that did not allow infants to be fed mother’s milk that was expressed outside of the hospital.(19) These findings led to a larger quality improvement initiative to enhance breastfeeding support, based by the Knowledge-to-Action framework.(20) The first step in the “knowledge to action cycle” involves identifying the gap between evidence-based recommendations and actual clinical practice.(20) Guided by the Neo-BFHI’s new international guidelines for optimizing breastfeeding support in neonatal units, the objective of this study was to explore breastfeeding support practices and related barriers and facilitators in the NICU at HUSVP.

## Methods

A multi-method descriptive design was used. Ethics approval for the study was obtained from all required institutional review boards. The NICU at HUSVF is a university-affiliated, tertiary-quaternary referral center in Medellin, Colombia with 14 intensive care, 24 intermediate care and 2 basic care beds. Members of the team involved in breastfeeding promotion and support included nurses, auxiliary nurses, physicians, physical therapists, social workers, and nutritionists. Data collection methods included:

Medical records review*.* To obtain preliminary baseline data on breastfeeding-related practices and outcomes in the HUSVP’s NICU, a retrospective review of medical records was conducted. A chart review form was adapted and piloted by the unit’s nurse managers and breastfeeding resource person to capture any breastfeeding-related data that were systematically documented in the infant’s medical records. The medical records of all infants born at < 37 weeks and consecutively discharged live from the NICU between June and September 2017 were reviewed. In the case of multiple births, only the first infant’s chart was reviewed, for a total of 51 infants. Data from the chart review were entered into an Excel spreadsheet and analyzed descriptively (means, ranges and/or proportions).

Survey of the unit’s breastfeeding practices*.* A key informant group interview was held with the NICU’s two nurse managers and breastfeeding resource person (an auxiliary nurse with advanced training in breastfeeding support), to complete a Spanish version of the *International Self-Assessment survey of policies and practices to protect, promote and support breastfeeding in neonatal wards* (12) The questionnaire was originally translated from English and reviewed for face validity by a team of neonatal care professionals from four different Spanish-speaking countries (Argentina, Peru, Chile and Spain). The questionnaire contains 63 indicators to assesses compliance with implementation of the Neo-BFHI’s Guiding Principles, Steps and Code, using three types of answer choices (Yes or No, and Likert scales ranging from “Never to Always”, or “None to All”). The first author (HA) administered a paper copy of the questionnaire to the group, and made hand-written notes of any pertinent verbal comments made by the key informants related to implementation of the Neo-BFHI’s indicators. The group interview took approximately two hours to complete.

Observations of NICU practices*.* To validate the questionnaire responses and collect more detailed information about the NICU’s setting, HA conducted three visits to the NICU to complete an observational checklist adapted from the questionnaire questions*.* The checklist included comment boxes to record any relevant observations related to the Neo-BFHI indicators, including any discrepancies between the questionnaire responses from the group interview and direct observations of practice.

Analysis. To synthesize the different sources of data, a word table was created to integrate the study findings within the broader categories of the Three Guiding Principles and Ten Steps. For each Guiding Principle and Step, the KI’s responses on the self-assessment survey and accompanying notes taken during the interviews were summarized in text by HA. Findings were then compared to data from the observational checklist and chart review to explore coherence between the different sources of data and identify any key discrepancies or contradictory findings. A summary text integrating information from all data sources was then created for each Guiding Principle and Step. All four authors participated in reviewing and validating the synthesized study findings.

## Results

### Chart review

Descriptive findings from the review of 51 infant charts are summarized in Table 1. The infant gestational age at birth ranged from 23.4 to 36.6 weeks, and a majority of infants (71%) were born via cesarean section. Of the 51 infant charts reviewed, almost all infants (98%) received their mother’s milk at some point during their hospitalization, and 96% were fed directly at the breast at least once prior to hospital discharge. However, 65% of infants received formula rather than breastmilk for their first oral feed. During their last 24 hours in the NICU, 65% of the infants were fed both formula and breastmilk and only 10% of the mothers intended to exclusively breastfeed following hospital discharge.

**Table 1:** Sociodemographic data, infant characteristics and infant feeding

Variable	***n* (%)**	Mean	Range
Multiple Birth			
Yes	12 (23.5)	-	-
No/not documented	39 (76.5)	-	-
Type of Birth			
Vaginal	15 (29.4)	-	-
Cesarean Section	36 (70.6)	-	-
Infant gestational age at birth (wks)	-	33.6	23.4-36.6
Infant weight at birth (gms)	-	1915	840-2960
Maternal age	-	24	14-44
Baby received mother’s milk during NICU			
hospitalization:			
Yes	50 (98.0)	-	-
Infant gestational age at first oral feed (wks)	-	34.6	26.2-41.2
Route of first oral feed:			
Bottle	43 (84.3)	-	-
Breast	7 (13.7)	-	-
Other	1 (2.0)	-	-
Type of first oral feed:		-	-
Colostrum/Breastmilk	16 (31.2)	-	-
Formula	33 (64.7)	-	-
Other/not documented	2 (4.0)	-	-
Breastfeeding initiated before NICU discharge		-	-
Yes	49 (96.1)	-	-
Infant gestational age at first documented breastfeed (wks)			
Infant weight at first breastfeed (gms)	-	35.6	31.3-41.2
Type of feeding in last 24 hrs of hospitalization:	-	2045	1555-3490
Breastmilk only	4 (7.8)		
Formula only	13 (25.5)	-	-
Formula + Breastmilk	33 (64.7)	-	-
Other	1 (2.0)	-	-
Mother intends to breastfeed exclusively			
Post NICU discharge			
Yes	5 (8.8)	-	-
No	46 (90.2)	-	-

### Compliance with the Neo-BFHI Recommendations

Findings from the self-assessment questionnaire, group interview and observations were integrated and summarized in descriptive notes related to implementation of each of the Neo-BFHI’s Guiding Principles and Steps.

### Guiding Principle 1: Staff attitudes must focus on the individual mother and her situation

According to the key informants (KIs), mothers on the unit were treated with sensitivity, empathy and respect for their maternal role by the clinical staff. However, due to lack of time and inadequate staffing, nurses were not always available to support mothers in making informed decisions about milk production, breastfeeding and infant feeding practices. Individualized breastfeeding support was available from the unit’s breastfeeding resource person (a nursing assistant who was responsible for the unit’s lactation room five days/week); nutrition students from the University of Antioquia; as well as weekly visits by volunteers from a community-based breastfeeding peer support program.

### Guiding Principle 2: The facility must provide family-centered care, supported by the environment

Although the term “family-centered care” was new for the KIs, they indicated that parents were always considered to be the most important persons in their infant’s life and were encouraged and supported to act as their infant’s primary caregiver. All staff were trained in IAMI (i.e., mother and baby-friendly care) principles and the organizational values promoted by the hospital staff included humanization of care and active participation of the patient and family in their health care experience. The KIs also responded that parents were encouraged to be involved in the care of their infant beginning within the first 24 hours of admission, and were kept informed frequently on their infant’s progress if they were not able to be with their infant in the first 24 hours. Questions related to the unit’s environment evaluated how the unit supported parental presence, as well as the level of light and noise in the NICU. Observations revealed that each incubator had only one plastic chair next to it (or no chair at all), limiting the ability of parents to rest comfortably together by their infant. Family members were not allowed to eat in the unit, but could eat within the same building. There were ten single rooms in the unit where staff were observed adjusting the lighting to the infant’s needs, whereas lighting could not be individualized in the shared rooms with multiple cribs. Despite Neo-BFHI recommendations that NICU noise levels should be kept low, both the questionnaire responses and observations suggested this indicator was rarely met. Parental privacy was limited as all patient rooms faced the unit’s central nursing station and had glass walls with no curtains, and there were not screens/dividers between incubators.

### Guiding Principle 3: The healthcare system must ensure continuity of care from pregnancy to after the infant’s discharge

The KIs reported that the NICU frequently worked with other units to coordinate breastfeeding support. For example, the unit’s breastfeeding resource person visited other parts pediatric units to support breastfeeding mothers and infants. The KIs also reported that during shift changeover and upon transfer of infants from one unit to another, nurses were always familiar with the nutritional requirements of the infant, if the mother was expressing milk and the infant’s clinical history, which was verified during observations of admissions and discharges on the unit. All parents received written information at hospital discharge that included guidance on breastfeeding at home, and when and where to seek medical attention if needed. As well, the chart review revealed that 94% of infants were registered at hospital discharge to receive follow-up care from community-based “Kangaroo Programs”.

*Step 1: Have a written breastfeeding policy that is routinely communicated to all staff.* Although the NICU has a breastfeeding policy in the form of Ten Steps for their IAMI program, it was not specifically adapted for the preterm/sick infant. The IAMI’s Ten Steps were available for staff and families on two visible posters in the unit. As part of their breastfeeding policy, the NICU followed the International Code of Marketing of Breast-Milk Substitutes.

*Step 2: Educate and train all staff in the specific knowledge and skills necessary to implement this policy.* According to the KIs, all staff working in the NICU receive a 2-hour course on the background and policies of the IAMI, within 6 months of working on the unit. Course content included the IAMI’s Ten Steps, the International Code of Marketing of Breast-milk Substitutes and how to support mothers who are not breastfeeding. However, contrary to the Neo-BFHI’s recommendations, staff did not receive any supervised clinical training related to breastfeeding support, and it was observed that nurses frequently referred mothers to the breastfeeding resource person in the lactation room when breastfeeding support was needed.

*Step 3: Inform hospitalized pregnant women at risk for preterm birth about breastfeeding.* The HUSVF provided in-hospital antenatal care to pregnant women who are at risk for preterm delivery or birth of a sick infant. The KIs reported that all pregnant women hospitalized at HUSVF received information about lactation and breastfeeding from the nurses on the antenatal unit, including a pamphlet that described the IAMI’s Ten Steps to successful breastfeeding.

*Step 4: Encourage early, continuous and prolonged mother-infant skin-to-skin contact/Kangaroo Mother Care.* According to the KIs, skin-to-skin contact with the mother is always attempted in the first hour following delivery for stable infants (regardless of gestational age or delivery type), following a routine pediatric evaluation performed immediately after delivery. The KIs further reported that the parents in the NICU were strongly encouraged to be skin-to-skin with their infants whenever possible. However, observations revealed an informal policy on the unit that parents could only hold their infant skin-to-skin if they were prepared to do so for at least two hours, due to concerns that frequent position changes were stressful for the infant. Although the KIs estimated that infants were placed skin-to-skin an average of 4-6 hours per day if their parents were present in the NICU, this length of skin-to-skin contact was not observed, despite the frequent presence of parents during the day shift.

*Step 5: Show mothers how to initiate and maintain lactation, and establish early breastfeeding with infant stability as the only criterion.* Education on breastfeeding was provided primarily by the unit’s breastfeeding resource person and the NICU nurses. The KIs reported that mothers who wish to breastfeed or give expressed breastmilk to their infants are instructed to initiate expression of milk within the first 6 hours following birth, and to express their milk at least 7 times in 24 hours. Although all mothers were shown how to manually express their milk, no written guidelines were available on the unit. Mothers were observed manually expressing milk or using the unit’s only hospital-grade electric breast pump in the lactation room during all three observational visits. The breast pump was operated and cleaned by the breastfeeding resource nurse when she was on the unit Monday-Friday from 7:00-17:00. However, mothers were not allowed to use the pump on their own outside of these hours, due to concerns about the risk of contamination if the pump was not cleaned properly. When the lactation room was not available, mothers had the option to express their milk manually into a cup at their infant’s bedside.

According to the KIs, infant stability (rather than gestational age or weight) was the only criteria used to determine the timing of initiation of feeding at the breast, as recommended by the Neo-BFHI. However, observations revealed a common belief among staff that infant sucking abilities were not developed enough for breastfeeding until 34 weeks of gestational age. The chart review confirmed that although 29% of infants received their first oral feeding before 34 weeks gestational age, the majority (84%) had their first oral feed via a bottle rather than at the breast. Although the chart review found that 96% of the infants initiated direct breastfeeding prior to hospital discharge, the mean age at first documented breastfeed ranged from 34.2-41.2 weeks gestational age, with a mean of 35.6 weeks.

*Step 6: Give no food or drink other than breastmilk, unless medically indicated.* The KIs reported that all newborns in the NICU were fed solely with human milk (expressed or directly at the breast) unless there was a justified medical reason to use a breast-milk substitute; their mother’s expressed milk was not available; or the mother chose not to breastfeed. However, the chart review revealed a high rate of formula use in the NICU, with only 8% of infants receiving nothing but breastmilk at the time of hospital discharge. Although mothers were encouraged to continue expressing their milk to maintain lactation, the NICU’s policies prohibited the use of breastmilk expressed outside of the unit due to potential risks of milk contamination. The infant’s supply of maternal milk was further restricted by limited access to the unit’s sole breast pump, and barriers to maternal visiting during the infant’s NICU hospitalization (e.g., having other children to care for at home).

*Step 7: Enable mothers and infants to remain together 24h hours/ day.* According to unit policies, parents were allowed to stay with their infant at all times day or night, except during sterile medical procedures. However, the unit did not have adequate facilities for mothers to rest comfortably in the unit, nor could they sleep elsewhere in the hospital once they were discharged home following childbirth. The KIs noted that due to the lack of rooming-in and comfort amenities in the NICU (such as a family lounge or eating area), family members rarely spent 24 hours a day on the unit. However, mothers and fathers were seen frequently on the unit between the hours of 7:00-17:00. Sleeping accommodations within a 10-minute walk from the hospital were also available for some parents of infants transferred from outside the city, upon referral by the social worker.

Step 8: Encourage Demand Breastfeeding or, When Needed, Semi-Demand Feeding as a Transitional Strategy for Preterm and Sick Infants. The KIs reported that the unit always followed recommended guidelines for transitioning infants from scheduled to demand feeding, including stopping routine supplementation and fixed feeding schedules once the infant is able to feed directly at the breast, and teaching parents how to recognize infant feeding cues. However, delaying the initiation of direct breastfeeding until 34 weeks gestational age and limited parental visiting were important barriers to complete transition to demand breastfeeding prior hospital discharge.

*Step 9: Use alternatives to bottle feeding at least until breastfeeding is well established, and use pacifiers and nipple shields only for justifiable reasons.* Questionnaire responses reported that expressed breast milk was always given to the infant by bottle or feeding tube, depending on the age and condition of the infant. The use of alternative oral feeding methods such as cup feeding, dropper, syringe, or spoon was not observed. Bottles were frequently used on the unit, since mothers were not present around-the-clock to breastfeed. However, pacifiers were not available and never used in the unit.

*Step 10: Prepare parents continued breastfeeding and ensure access to support after hospital discharge* **.** The KIs reported that since the NICU discharged all infants directly to their homes, discharges were always planned in collaboration with the family and the primary care services located in their neighborhood. The NICU discharge plan was supposed to include early post-discharge follow-up by community-based professionals trained in infant feeding, if the mothers’ breastfeeding goals were not established prior to NICU discharge. However, the chart review found that only 10% of the mothers planned to exclusively breastfeed following discharge, although 73% were still providing their infant with some breastmilk at hospital discharge.

### Compliance with the International Code of Marketing of Breast-milk Substitutes.

The KIs reported that the HUSVP (including the NICU) adhered to the International Code of Marketing of Breast-milk Substitutes by refusing free or low-cost supplies of breast-milk substitutes, refraining from advertising or providing samples of breast-milk substitutes, bottles, and pacifiers in the unit, and by keeping infant formula and prepared bottles out of sight in the hospital’s milk lab*.* Located in another building from the NICU*,* the milk lab was where all infant milk feedings were prepared, bottled, and labelled by the nutritionist and support staff, for distribution around the hospital. According to the KIs, most staff on the unit understand why it was important not to give any free samples or promotional materials from formula companies to mothers, and this practice was not observed on the unit.

## Discussion

As the first step in a quality improvement initiative, this project used the new Neo-BFHI recommendations to identify gaps in breastfeeding support in the NICU at the HUSVP in Medellin, Colombia. This is the first paper we are aware of to document NICU breastfeeding support practices in a NICU in Colombia, contributing to scant data on neonatal nursing care in Latin America. Completing the Neo-BFHI’s self-assessment questionnaire was the NICU’s first exposure to the Neo-BFHI’s Guiding Principles and Ten Steps, and the unit’s leaders were eager to examine their care practices in light of these new recommendations.

Several discrepancies were noted between the questionnaire responses provided by the unit’s leaders, and infant feeding practices observed on the unit or documented in the infant’s chart review. Collecting both qualitative and quantitative data during improvement projects is critical for evaluating care quality.(21) For example, the WHO’s assessment process for Baby-Friendly accreditation recommend using multiple sources of data to assess compliance with the BHFI, including interviews with managers, staff, and patients; observations of local care practices; the facility’s breastfeeding statistics and a review of documents and training curriculum related to BFHI policies.(22) Findings from the chart review also revealed a lack of systematic documentation of practices associated with breastfeeding exclusivity and duration, such as parent/infant skin-to-skin contact time, breastfeeding attempts, maternal pumping volumes and bottle use. Integrating such key clinical indicators into the unit’s documentation systems would facilitate data collection for ongoing quality monitoring and benchmarking of breastfeeding support practices.(16)

The Neo-BFHI self-assessment questionnaire identified many local facilitators for breastfeeding success in the NICU, including a strong family-centered care philosophy, a full-time breastfeeding resource person dedicated to the unit, and an almost universal breast milk feeding initiation rate of 98%. However, findings revealed several potential barriers to breastfeeding support that could be targeted for quality improvement. For example, although parents had unlimited access to their infants, a lack of facilities to comfortably support parental presence at their infant’s bedside limited the duration of parental visiting. Mother-infant separation in the NICU is well-recognized as a key barrier to the establishment of successful breastfeeding, limiting opportunities for mother-infant bonding (e.g., via skin-to-skin contact) and the transition to direct breastfeeding.(18) An international questionnaire of Neo-BFHI compliance in 917 neonatal units from 36 countries also revealed lower implementation of Step 7, highlighting the challenges of enabling mother-infant contact in the neonatal intensive care environment.(11) Small, low-cost improvements such as providing screens for privacy, having more comfortable (and preferably, reclining) chairs at the bedside or creating a space for parents in or near the NICU to rest and eat, may help prolong maternal visiting and facilitate breastfeeding.

The NICU’s policy of not allowing mothers to transport breastmilk expressed outside of the hospital also limited their infant’s access to breast milk, and has been previously associated with maternal distress and decreased lactation in Brazil.(18) To comply with the Neo-BFHI’s recommendation of feeding infants only breastmilk, NICUs that lack rooming-in facilities require the necessary teaching tools, policies and resources to support regular maternal expression and safe storage, handling and transport of milk (or access to donor milk).(15) Despite the critical importance of breastfeeding for the health and development of preterm and ill infants, application of the Neo-BFHI’s evidence-based guidelines may be challenging in low-resourced settings where families may lack proper sanitation, milk expression equipment or the means to regularly visit their hospitalized infant.

Limitations. As this study only assessed breastfeeding support practices in one NICU, findings may not be generalizable to other settings. Another limitation is that the self-assessment questionnaire was completed by the unit’s managers, who may have been subject to a positive response bias. However, this was addressed by collecting more objective data from observations and chart reviews. In future studies, bedside care providers may provide a more realistic assessment of local breastfeeding support practices than busy managers, who may be more removed from clinical activities. Additionally, parents’ perceptions of unit compliance with the Neo-BFHI’s Ten Steps and Guiding Principles would contribute to a more comprehensive understanding of barriers and facilitators to NCU breastfeeding support.

Conclusion. The Neo-BFHI self-assessment questionnaire combined with chart reviews and observations allowed for the comprehensive assessment of evidence-based practices to support breastfeeding in a Colombian NICU. Findings revealed a high value for breastfeeding and a family-centered approach to care in the NICU. Key challenges to sustaining breastfeeding in the NICU that could be prioritized for practice improvement included a lack of facilities for supporting parental presence, barriers to expression and provision of mother’s milk, and a high rate of bottle-feeding with formula. 
